# Serum aminoterminal proctype natriuretic peptide in girls with idiopathic central precocious puberty during GNRHA treatment

**DOI:** 10.1186/1687-9856-2013-S1-O24

**Published:** 2013-10-03

**Authors:** Qiu-li Chen, Hua-mei Ma, Zhe Su, Yan-hong Li, Hong-shan Chen, Min-lian Du

**Affiliations:** 1 The first Affiliated Hospital of Sun Yat-Sen University, Guangzhou, China

## 

The mechanism of linear growth reduction during GnRHa treatment in central precocious puberty has not been elucidated.

## Aim

To investigate the pattern of serum amino-terminal proC-type natriuretic peptide (NT proCNP) in healthy girls throughout puberty, and the changes of serum NT proCNP in girls with idiopathic central precocious puberty (ICPP) before and during gonadotropin-releasing hormone analog(GnRHa) therapy.

## Methods

Serum levels of E_2_, NT proCNP, insulin like growth factor 1(IGF1), NMID Osteocalcin(OC) and carboxy-terminal cross-linking telopeptide of type I collagen (β-CrossLaps) were measured in healthy 57 girls at different pubertal stages, and in 13 girls with ICPP at the beginning and the end of 6^th^ month and 12^th^ month of GnRHa treatment. Height velocities of the 13 ICPP girls in each 6 months before and after GnRHa treatment was calculated.

## Results

Serum NT proCNP level increases as the progress of pubertal development and peaks at the late puberty (P<0.01), paralleling with serum E_2_ and IGF1 levels, like with the pattern of height velocity. All of serum NT proCNP, Osteocalcin and β-CrossLaps level decrease significantly in ICPP girls at the end of 6^th^ months of GnRHa therapy(P<0.01 or P<0.05), and remain the same low level at the end of 12^th^ month of GnRHa. Different from the aboved markers, serum IGF1 level remains high before and during GnRHa treatment despite growth deceleration.

## Conclusions

Linear growth reduction in girls with ICPP treated with GnRHa is due at least in part to decreased CNP mediated long bone growth after estrogen inhibition. Serum NT proCNP can be used as a biological marker of long bone growth indicating the activity of epiphyseal growth plate.

